# Predicting machine's performance record using the stacked long short‐term memory (LSTM) neural networks

**DOI:** 10.1002/acm2.13558

**Published:** 2022-02-16

**Authors:** Min Ma, Chenbin Liu, Ran Wei, Bin Liang, Jianrong Dai

**Affiliations:** ^1^ Department of Radiation Oncology National Cancer Center/National Clinical Research Center for Cancer/Cancer Hospital Chinese Academy of Medical Sciences and Peking Union Medical College Beijing China; ^2^ Department of Radiation Oncology National Cancer Center/National Clinical Research Center for Cancer/Cancer Hospital and Shenzhen Hospital, Chinese Academy of Medical Sciences and Peking Union Medical College Shenzhen China

**Keywords:** long short‐term memory networks (LSTM), predictive time series, quality control, radiotherapy

## Abstract

**Purpose:**

The record of daily quality control (QC) items shows machine performance patterns and potentially provides warning messages for preventive actions. This study developed a neural network model that could predict the record and trend of data variations quantitively.

**Methods and materials:**

The record of 24 QC items for a radiotherapy machine was investigated in our institute. The QC records were collected daily for 3 years. The stacked long short‐term memory (LSTM) model was used to develop the neural network model. A total of 867 records were collected to predict the record for the next 5 days. To compare the stacked LSTM, the autoregressive integrated moving average model (ARIMA) was developed on the same data set. The accuracy of the model was quantified by the mean absolute error (MAE), root‐mean‐square error (RMSE), and coefficient of determination (*R* ^2^). To validate the robustness of the model, the record of four QC items was collected for another radiotherapy machine, which was input into the stacked LSTM model without changing any hyperparameters and ARIMA model.

**Results:**

The mean MAE, RMSE, and$\;R^{2}$ with 24 QC items were 0.013, 0.020, and 0.853 in LSTM, while 0.021, 0.030, and 0.618 in ARIMA, respectively. The results showed that the stacked LSTM outperforms the ARIMA. Moreover, the mean MAE, RMSE, and$\;R^{2}$ with four QC items were 0.102, 0.151, and 0.770 in LSTM, while 0.162, 0.375, and 0.550 in ARIMA, respectively.

**Conclusions:**

In this study, the stacked LSTM model can accurately predict the record and trend of QC items. Moreover, the stacked LSTM model is robust when applied to another radiotherapy machine. Predicting future performance record will foresee possible machine failure, allowing early machine maintenance and reducing unscheduled machine downtime.

## 1 INTRODUCTION

Linear accelerators (Linacs) undergo daily quality control (QC) items to ensure that radiation treatments are delivered safely and accurately, and that they meet the quality and safety criteria of AAPM TG 142.^1^ Daily QC items are normally performed by the radiotherapist using a conventional QC instrument or phantom.

These sequential sets of the record of daily QC items measured over successive days can be considered time series. Therefore, in the context of time‐series predictive modeling, the question of predicting the future record and trend has been raised.^2^ Traditionally, statistical modeling techniques like to autoregressive integrated moving average model (ARIMA)^3^ and their variations (autoregressive model (AR), moving average model (MA), autoregressive moving average model (ARMA)),^4^, ^5^, ^6^ only capture the linear elements of the time series and may not be sufficient for the daily QC record. Nonlinear time series are best analyzed using recurrent neural network (RNN). However, RNN is difficult to deal with long time series.^7^ Therefore, the long short‐term memory (LSTM) network is proposed to tackle the forgetting problem,^8^ a type of RNN. LSTM has shown good performance in various fields (finance, public transportation, astronomy, environmental science, and medicine).^7^ In the previous studies of the predictive model development about daily QC in Linac, Li et al.^2^ used artificial neural networks (ANNs), and ARMA on 5‐year daily beam QC record, which showed ANN had better prediction performance than ARMA. Puyati et al.^9^ used statistical process control and ARIMA to forecast QC. However, there is no good performance to predict QC record and trend in Linac, and time lags exist in the predictive model.

The daily QC record is used to track the Linac's long‐term stability when processing large quantities of record. With these records, medical physicists could calibrate the baseline and monitor the Linac's state to predict variation cycles and take preventive actions. To understand the underlying structure and functions that produce the observed QC tests, an appropriate modeling tool is needed to extract and analyze the longitudinal record of daily QC items and predict future trends.

In this study, a generalized LSTM model was developed to predict the record and trend of daily QC items for two radiotherapy machines. Additionally, this study emphasized on discovering the common behaviors of the Linac performance so that physicists can be more confident in predicting the machine's future behavior and taking action in a planned way before the tolerance level is reached. Finally, to compare and provide context for our results, we also developed a prediction model with ARIMA on the same data set.

## 2 METHODS AND MATERIALS

### 2.1 Data acquisition

The Varian Edge Linacs (Varian Medical Systems, Inc., Palo Alto, CA) was installed and commissioned in our institute in May 2017. The machine performance check (MPC) is equipped for daily QC tests on the Edge Linac. MPC is a fully integrated KV and MV image‐based tool to examine and evaluate the machine's performance.^10^

On every workday, MPC process ran about 5 min by radiation technician. Twenty‐four QC items for 6 MV X‐ray were run, including isocenter, collimation, gantry, and couch tests. The QC tests are highly automated, and the user only should set up the IsoCal phantom and bracket on the treatment couch, then beam on for the predetermined energy. MPC application has been evaluated as a Linac daily QC tool by some investigators.^10^, ^11^, ^12^, ^13^, ^14^ In this study, the records of 24 QC items based on MPC were collected at our institution using Varian Edge for more than 3 years, from August 2017 to October 2020. A total of 867 data were collected to predict the record for the next 5 days.

### 2.2 Data preprocessing

Preprocessing data are a significant step before building a model. In this study, performing data preprocessing included cleaning, interpolation, normalization, and data split. The duplicate data were deleted at the starting point. Cubic interpolation was used to double the amount of data to improve the prediction accuracy. The data were normalized for the model, ranging from −1 to 1. The data were divided into three sets: 70% for training, 15% for validation, and 15% for testing. The training set and the validation set were used to train the model with different hyperparameter combinations (see Section 2.3.2). The testing set was used to assess the performance of the model with the optimal hyperparameter combination.

### 2.3 Building LSTM network

#### 2.3.1 LSTM network

**FIGURE 1:**
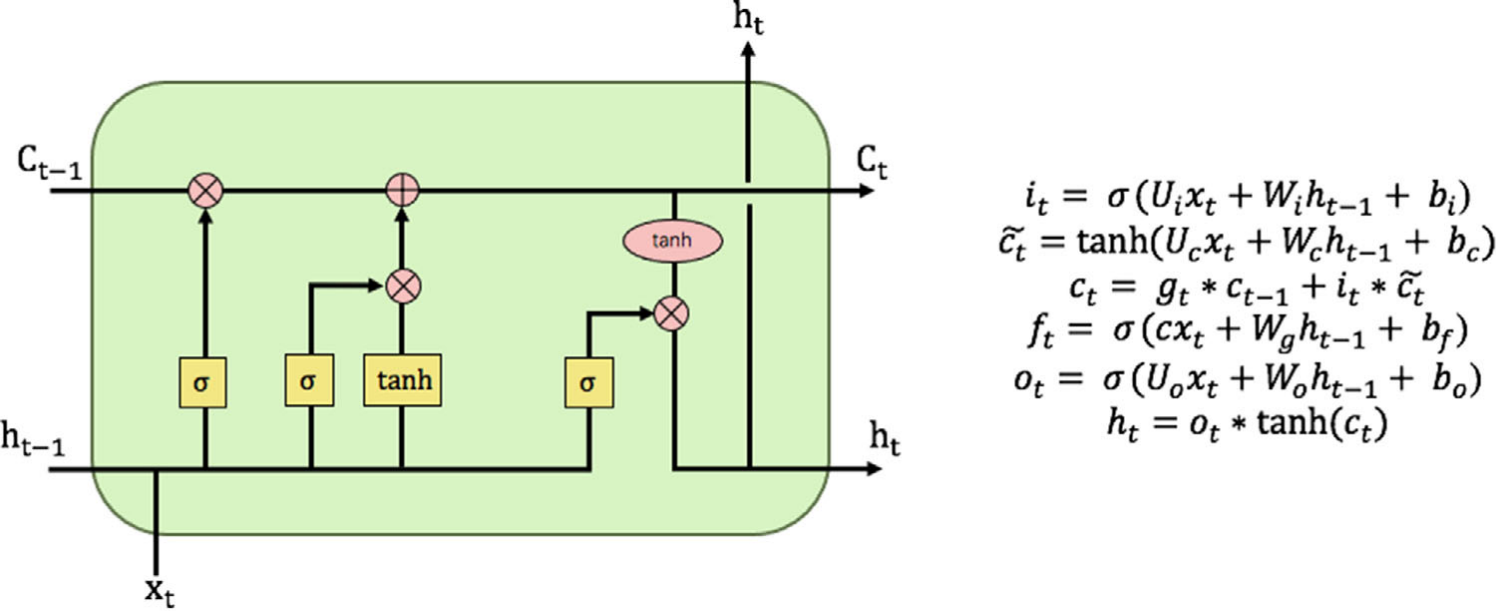
The structure of long short‐term memory (LSTM) as described by Varsamopoulos.^29^ Input gate ($i_{t}$), input module gate ($\tilde{c_{t}}$), forget gate ($f_{t}$), and output gate ($o_{t}$). b is bias vectors, $c_{t}\;$is cell state, $h_{t}$ is the hidden state, and σ is the sigmoid activation function

LSTM is very powerful in solving sequence prediction problems because it can store previous information,^15^ which is essential to predict the future record and trend of daily QC items. Through the standard recurrent layer, self‐loops, and the internal unique gate structure, the LSTM network effectively improves the forgetting and gradient vanishing problem existing in the traditional RNN.^8^ Besides, LSTM can learn to make a one‐shot multi‐step prediction useful for predicting the time series. An LSTM neural network unit combines four gates: an input gate, a cell state, a forgotten gate, and an output gate (Figure 1).^16^ The forget gate is used to determine which messages pass through the cell, then enter the input gate, which decides how many new messages to add to the cell state, and finally decide the output message through the output gate.^17^

**FIGURE 2:**
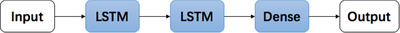
The stacked long short‐term memory (LSTM) architecture

The original LSTM model is comprised of a single hidden LSTM layer followed by a standard feedforward output layer. The stacked LSTM is an extension to this model that has multiple hidden LSTM layers where each layer contains multiple memory cells.^7^ The stacked LSTM hidden layers make the model deeper, more accurately earning the description as a deep learning technique. It is the depth of neural networks that are attributed to the approach's success on various challenging prediction problems.^18^ The stacked LSTM is now a stable technique for challenging sequence prediction problems.^19^ An LSTM model with many LSTM layers is a stacked LSTM architecture (Figure 2).^20^ An LSTM layer above provides a sequence output rather than a single value output to the LSTM layer below. Specifically, one output per input time step is one output time step for all input time steps. Therefore, in this study, the stacked LSTM was selected.

For the ARIMA, there are three critical parameters in ARIMA: p (the past value used to predict the next value), q (past prediction error used to predict future values), and d (order of differencing).^21^, ^22^ ARIMA parameter optimization requires much time. Therefore, in this study, ARIMA selected the combination (5, 1, 0).

#### 2.3.2 Model training

The experiment's LSTM model was built on the Keras API package (TensorFlow2.0) in Python 3.6 settings (Python Software Foundation, Wilmington, DE). In this study, networks with two LSTM layers were investigated. The loss value was evaluated by the root‐mean‐square error (RMSE). The activation functions used the rectified linear units (Relu) function. A greedy coordinate descent method^23^ was employed to find the optimal hyperparameter of the model.

The length of time lags, the optimizers, the learning rates, the number of epochs, the number of hidden units, and the batch sizes were among the tuning parameters. First, we sought to find the optimal length of time lags when the optimizer was Adam, the learning rate was 0.01, the number of epochs was 150, the number of hidden units was 50, and the batch sizes were 32. Subsequently, we determined the type of optimizer with the optimal length of time lags. Next, the appropriate learning rate was determined by comparing results from various learning rates. Then, we sought to find the optimal number of epochs and hidden units in turn. Lastly, to determine the optimal batch size, a similar comparison was performed. The batch size was adjusted to avoid errors from memory shortage. By testing the parameter values of different combinations, and the model suitable for the data was finally found.

Hyperparameters selection and optimization play an important role in obtaining superior accuracy with the LSTM network.^24^ The validation set's mean absolute error (MAE) was used to evaluate the model's performance for each parameter combination. The investigated hyperparameters and their range are listed in Table 1.

**TABLE 1:** The summary of long short‐term memory (LSTM) hyperparameters investigated in this study, and the recommended configurations, and the impact level of each parameter

Hyperparameters	Range	Recommended configuration	Impact
Length of time lags	(1, 5, 10, 15, 20, 30, 50)	15	Middle
Optimizer	{Adam, RMSprop, Adagrad, Adadelta}	Adam	High
Learning rate	(0.0001, 0.001, 0.01, 0.1)	0.001	High
Number of epochs	(50, 100, 150, 200, 250, 300)	300	Low
Number of hidden units per layer	(10, 30, 50, 70, 100)	50	Middle
Batch size	(8, 16, 32, 64, 128, 256)	32	Low

#### 2.3.3 Evaluation of predictive accuracy

To evaluate the error between the predicted and observed values in the testing set, the RMSE, MAE, and coefficient of determination ($R^{2}$) were selected.

### 2.4 The trend lines

The trend lines were used to analyze the trend of Linac operating status and thereby help medical physicists decide whether to take preventive actions. The stacked LSTM model was applied to predict the next 5‐day record of 24 QC items in this study. The trend lines were plotted by the polynomial fit from five‐step‐head predictive values. If the trend line exceeds the tolerance value of the QC item, preventive measures need to be taken.

### 2.5 Effectiveness evaluation

To evaluate the performance of the stacked LSTM model, the record of daily QC items was investigated for another radiotherapy machine. The evaluation data set was collected from the Novalis Tx (Varian, Palo Alto, CA) machine between May 2020 and November 2021. A total of 415 records were collected. On every workday, the daily QC item was measured by the technician using the QUICKCHECK^webline^ (PTW, Freiburg, Germany) phantom. The records of four QC items—output constancy, beam symmetry along gun target direction (GT) and left‐right direction (LR), and beam quality factor (BQF) for 6 MV X‐ray—were selected. The records of these four QC items were input into the stacked LSTM model without changing any hyperparameters chosen for Section 2.3.2 and ARIMA model. Then predictive accuracy was evaluated by the RMSE, MAE, and $R^{2}$ between the predicted and observed values. Finally, comparison of predicted and observed record of four QC items based on Quickcheck was plotted. Those aimed to observe the accuracy of the prediction and assess the robustness of the stacked LSTM model.

## 3 RESULTS

### 3.1 Hyperparameter tuning in LSTM

**FIGURE 3:**
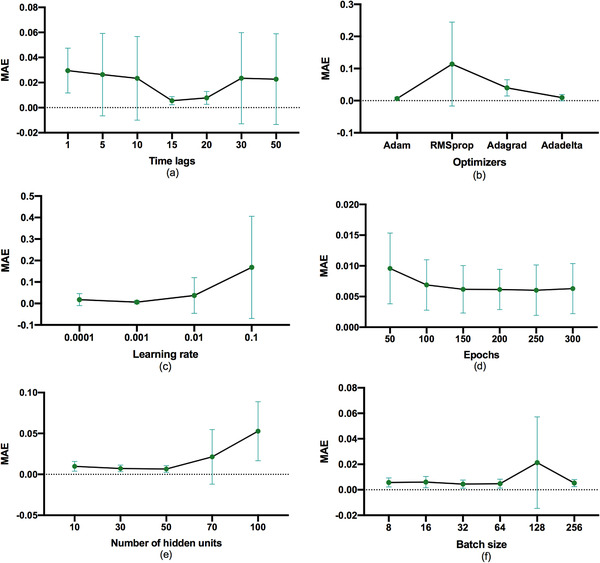
The mean absolute error (MAE) of predicted data (mean value in green, 95% confidence interval (CI) in blue) with different values of (a) the length of time lags, (b) the optimizers, (c) the learning rates, (d) the number of epochs, (e) the number of hidden units, and (f) the batch sizes

Figure 3 shows the MAE (in a relative unit) as a function of time lags, optimizers, learning rates, epochs, hidden units, and batch sizes. The optimal hyperparameter value is summarized in Table 1. Among them, the learning rate had the greatest impact on the model. The best performance was set to 0.001 of the learning rates, and the worst was set to 0.1, causing up to 0.039 difference in relative MAE. Furthermore, the type of optimizer had the second greatest impact on the model. In comparison, the length of time lags and the number of hidden units demonstrated only a modest impact on the model's predictive performance. Finally, the number of epochs and the batch size showed little impact on the predictive accuracy.

### 3.2 Predictive performance evaluation

**FIGURE 4:**
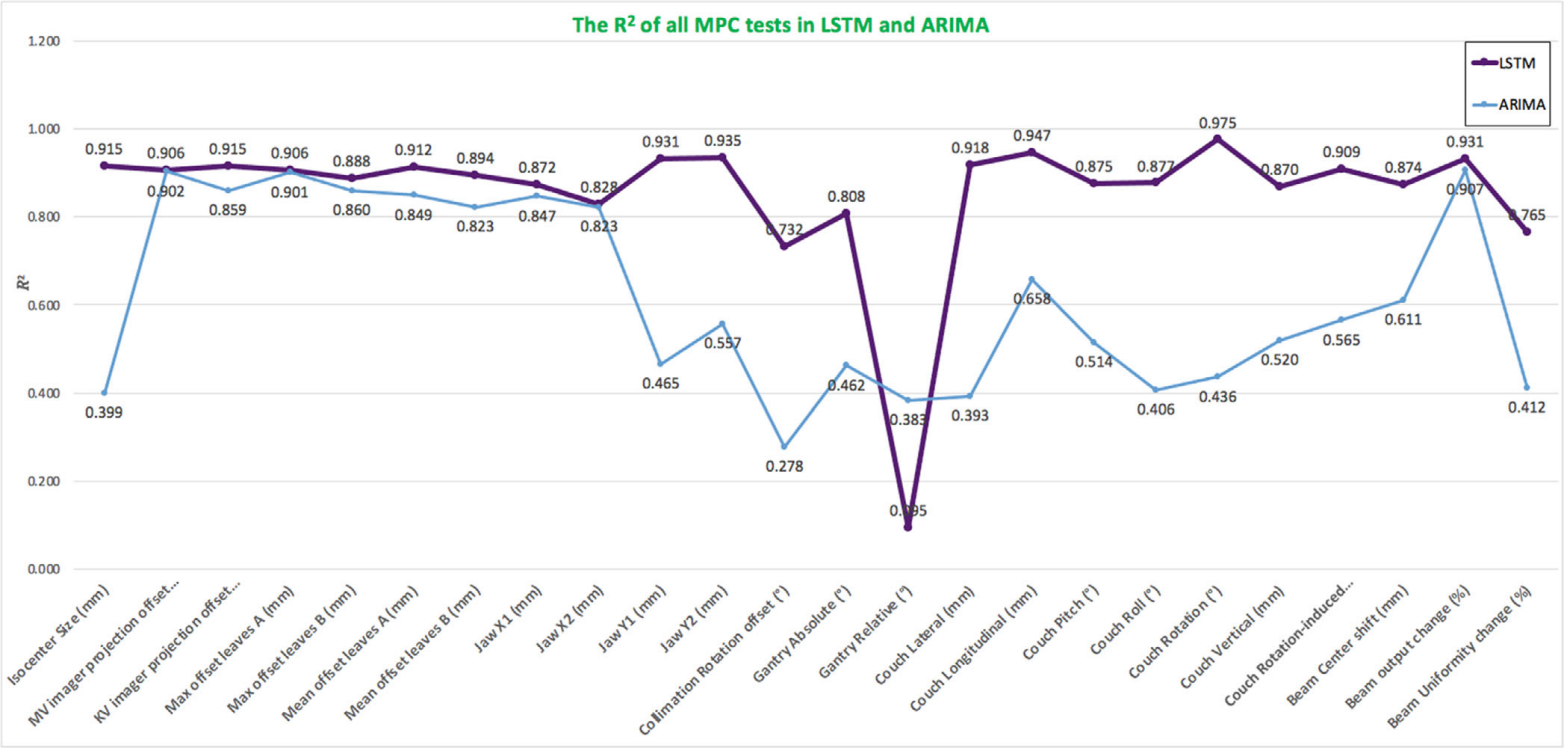
Comparison graph of model performance for 24 quality control (QC) items based on machine performance check (MPC) in the coefficient of determination ($R^{2}$). The purple line means long short‐term memory (LSTM), and the blue line meansautoregressive integrated moving average model (ARIMA)

Table 2 shows the performance of the stacked LSTM model in predicting daily QC items based on MPC using the optimal hyperparameter and ARIMA. The mean MAE, RMSE, and $R^{2}$ with 24 MPC items were 0.013, 0.020, and 0.853 in LSTM, while 0.021, 0.030, and 0.618 in ARIMA, respectively. LSTM performed better than ARIMA in 23 MPC items with the smaller MAE value, smaller RMSE value, and higher $R^{2}$, except for gantry relative (LSTM: MAE = 0.006, RMSE = 0.007, and $R^{2}$ = 0.095; ARIMA: MAE = 0.004, RMSE = 0.006, and $R^{2}$ = 0.383). The best predictive performance of LSTM was couch rotation (MAE = 0.001, RMSE = 0.004, and $R^{2}$ = 0.975), but the worst was gantry relative (MAE = 0.006, RMSE = 0.007, and $R^{2}$ = 0.095). Additionally, Figure 4 shows the comparison of model performance in terms of the coefficient of determination ($R^{2}$). The $R^{2}$ value of LSTM is higher point than ARIMA in Figure 4, except for the $R^{2}$ value of gantry relative. In general, the stacked LSTM outperformed the ARIMA.

**TABLE 2:** The accuracy of the stacked long short‐term memory (LSTM) and autoregressive integrated moving average model (ARIMA) model for 24 quality control (QC) items based on machine performance check (MPC)

MPC test		MAE		RMSE		*R*2	
Categories	QC items	LSTM	ARIMA	LSTM	ARIMA	LSTM	ARIMA
Isocenter	Size (mm)	0.002	0.006	0.003	0.008	0.915	0.399
	MV imager projection offset (mm)	0.010	0.013	0.020	0.020	0.906	0.902
	KV imager projection offset (mm)	0.013	0.015	0.018	0.024	0.915	0.859
Collimation	Max offset leaves A (mm)	0.010	0.009	0.012	0.013	0.906	0.901
	Max offset leaves B (mm)	0.008	0.008	0.010	0.012	0.888	0.860
	Mean offset leaves A (mm)	0.007	0.009	0.010	0.013	0.912	0.849
	Mean offset leaves B (mm)	0.006	0.008	0.008	0.011	0.894	0.823
	Jaw X1 (mm)	0.012	0.013	0.018	0.019	0.872	0.847
	Jaw X2 (mm)	0.011	0.013	0.019	0.019	0.828	0.823
	Jaw Y1 (mm)	0.017	0.044	0.022	0.062	0.931	0.465
	Jaw Y2 (mm)	0.017	0.043	0.022	0.057	0.935	0.557
	Rotation offset (°)	0.030	0.047	0.039	0.064	0.732	0.278
Gantry	Absolute (°)	0.003	0.006	0.005	0.008	0.808	0.462
	Relative (°)	0.006	0.004	0.007	0.006	0.095	0.383
Couch	Lateral (mm)	0.005	0.012	0.006	0.016	0.918	0.393
	Longitudinal (mm)	0.004	0.010	0.006	0.014	0.947	0.658
	Pitch (°)	0.001	0.002	0.002	0.003	0.875	0.514
	Roll (°)	0.002	0.004	0.002	0.005	0.877	0.406
	Rotation (°)	0.001	0.002	0.001	0.004	0.975	0.436
	Vertical (mm)	0.008	0.016	0.011	0.021	0.870	0.520
	Rotation‐induced couch shift (mm)	0.008	0.017	0.011	0.024	0.909	0.565
Beam	Center shift (mm)	0.014	0.027	0.021	0.037	0.874	0.611
	Beam output change (%)	0.069	0.098	0.120	0.139	0.931	0.907
	Uniformity change (%)	0.054	0.082	0.078	0.129	0.765	0.412
Mean		0.013	0.021	0.020	0.030	0.853	0.618

Abbreviations: MAE, mean absolute error; RMSE, root‐mean‐square error.

**FIGURE 5:**
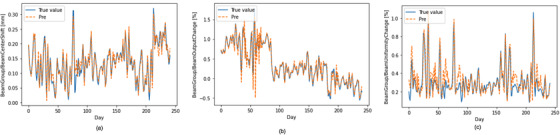
Comparison of predicted and observed beam quality control (QC) records, including (a) beam center shift, (b) beam output change, and (c) beam uniformity change using the stacked long short‐term memory (LSTM) model with the optimal hyperparameters in testing data

Figure 5 depicts three representative cases (beam center shift, beam output change, and beam uniformity change) of the observed versus the predicted curves using the stacked LSTM model with the optimal hyperparameter combination in testing data.

### 3.3 The trend lines

**FIGURE 6:**
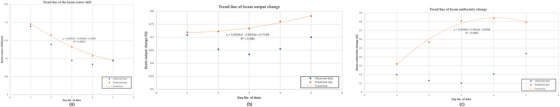
An example of the trend line to detect (a) the beam center shift, (b) beam output change, and (c) beam uniformity change

The weekly trend line for the beam is shown in Figure 6. All predictive values were within the tolerance. The trend was that the beam center shift drops but remains at normal‐stage levels. The trend of the beam output change and beam uniformity change rose, located in the normal range. This provided the opportunity to adjust the machine.

### 3.4 Validation of effectiveness

Table 3 shows the performance of the stacked LSTM model in predicting four QC items based on Quickcheck without changing any hyperparameters and ARIMA. The mean MAE, RMSE, and $R^{2}$ with four QC items were 0.102, 0.151, and 0.770 in LSTM, while 0.162, 0.375 and 0.550 in ARIMA, respectively.

**TABLE 3:** The accuracy of the stacked long short‐term memory (LSTM) and autoregressive integrated moving average model (ARIMA) model for four quality control (QC) items based on Quickcheck

Quickcheck		MAE		RMSE		*R*2	
Categories	QC items	LSTM	ARIMA	LSTM	ARIMA	LSTM	ARIMA
Beam	Output dose	0.231	0.458	0.373	1.223	0.741	0.309
	Symmetry GT	0.095	0.096	0.121	0.145	0.792	0.691
	Symmetry LR	0.072	0.075	0.094	0.103	0.704	0.641
	Beam quality factor	0.011	0.020	0.017	0.028	0.845	0.561
Mean		0.102	0.162	0.151	0.375	0.770	0.550

Abbreviations: GT, gun target direction; LR, left‐right direction; MAE, mean absolute error; RMSE, root‐mean‐square error.

**FIGURE 7:**
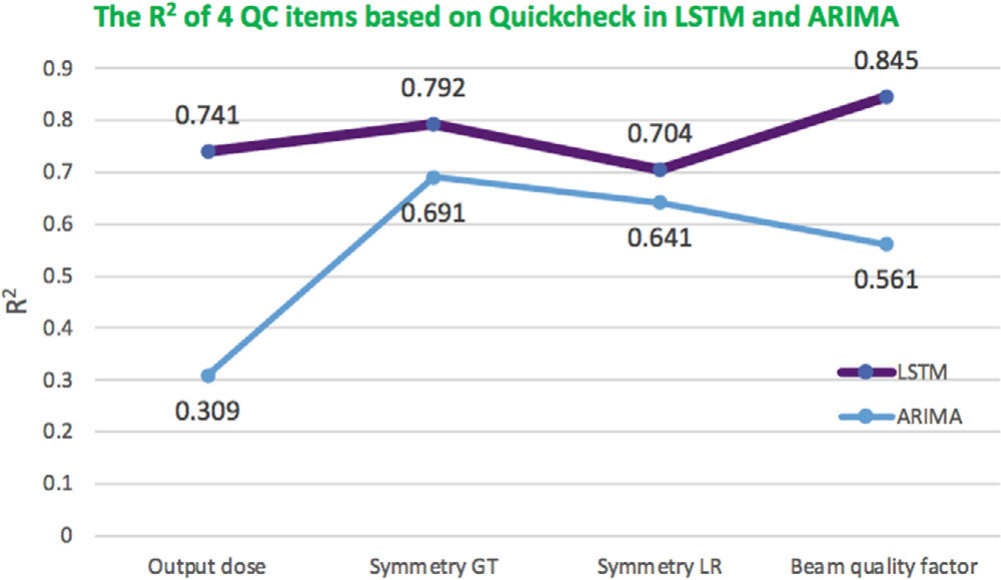
Comparison graph of model performance for four quality control (QC) items based on Quickcheck in the coefficient of determination ($R^{2}$). The purple line means long short‐term memory (LSTM), and the blue line means autoregressive integrated moving average model (ARIMA)

**FIGURE 8:**
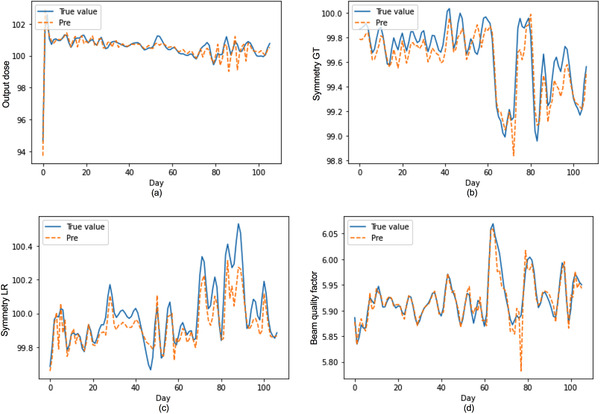
Comparison of predicted and observed record of four quality control (QC) items based on Quickcheck, including (a) output dose, (b) symmetry gun target direction (GT), (c) symmetry left‐right direction (LR), and (d) beam quality factor using the stacked long short‐term memory (LSTM) model without changing any hyperparameters

The $R^{2}$ value of LSTM is higher point than ARIMA for four QC items in Figure 7. Figure 8 depicts four QC items (output dose, Symmetry GT, Symmetry LR, and Beam quality factor) of the observed versus the predicted curves using the stacked LSTM model without changing any hyperparameters based on Quickcheck.

## 4 DISCUSSION

**FIGURE 9:**
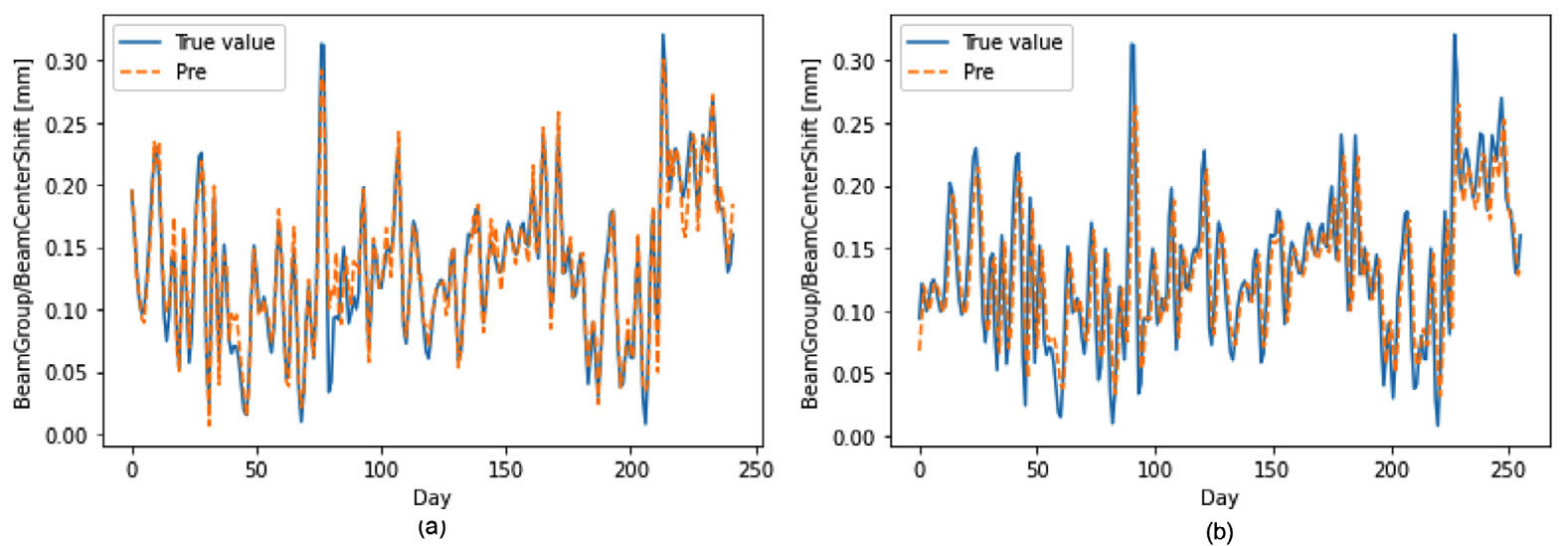
The predictive performance of the beam center shift based on machine performance check (MPC) with (a) the length of time lags = 15 ($R^{2}=\;0.874$), and (b) the length of time lags = 1 ($R^{2}=\;0.603$) in the stacked long short‐term memory (LSTM) model

This study demonstrates the need to tuning the hyperparameters using a deep LSTM model for daily QC items to obtain good predictive results. The learning rate determines how fast your neural net “learns.” The challenge of using a learning rate is that their hyperparameters must be defined in advance, and they depend heavily on the type of model and problem. Adaptive gradient descent algorithms (Adagrad, Adadelta, RMSprop, Adam) provide a heuristic approach without requiring expensive work to manually tuning hyperparameters for the learning rate.^25^ According to the MAE value (Figure 3), Adam and learning rate setting to 0.001 was recommended to use in the stacked LSTM model. Besides, when adjusting the different lengths of time lags, the LSTM predictive effect will be delayed (Figure 9). $R^{2}$ value of the beam center shift is 0.603 (the lengths of time lag = 1), while $R^{2}$ value of the beam center shift is 0.874 (the lengths of time lag = 15). Lag observations for a univariate time series can be used as time lags for an LSTM model, which can improve forecast performance.

**FIGURE 10:**
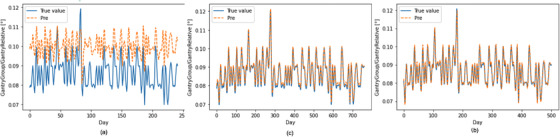
The predictive performance of the gantry relative to (a) two times cubic interpolation ($R^{2}=\;0.095)$, (b) four times cubic interpolation ($R^{2}=\;0.846$), and (c) six times cubic interpolation ($R^{2}=\;0.978$) in the stacked long short‐term memory (LSTM) model

This is the first study to implement a stacked LSTM model for daily QC record prediction to the best of our knowledge. It is one of the first few attempts to develop and evaluate a single generic stacked LSTM model. The stacked LSTM model allowed connections through time and provided a way to feed the hidden layers from previous steps (long‐term and short‐term) as additional inputs to the next stage, in contrast to earlier studies that only focused on studying the power of ANN.^2^ The stacked LSTM is effective at predicting daily MPC record. However, the generic stacked LSTM is poor in predicting the record of the gantry relative with two times cubic interpolation. In Figure 8a, the predictive range is significantly shifted up and slightly delayed. According to Wang et al. study^26^ about “Why are the ARIMA and SARIMA not sufficient?,” we guess that LSTM predictive performance is related to the signal frequency. Interpolation reduces high‐frequency signals and can greatly improve the predictive ability of the stacked LSTM model. Therefore, we try four times cubic interpolation and six times cubic interpolation in the stacked LSTM model, which significantly improves the accuracy performance (Figure 10b,c). The predictive performance of the gantry relative with six times cubic interpolation is great ($R^{2}$ = 0.978) in the stacked LSTM model.

For all daily MPC tests, the predicted record locates within the clinical tolerances (AAPM TG‐142),^1^ providing a window of opportunity to prevent the performance issue in advance. However, in the actual situation, besides keeping parameters within the tolerance, a clinical physicist should monitor trends in the machine performance^27^ and to know when the Linac needs to be maintained, thereby reducing the chance of Linac downtime. Here, a five‐step‐ahead prediction is appropriate to provide trends in Linac status. If the data point is within the tolerance, the newly entered data can be monitored.

To illustrate the robustness of the LSTM model, the record of four QC items based on Quickcheck in another Varian Linac was applied. LSTM performed better than ARIMA in four QC items with the smaller MAE value, smaller RMSE value, and higher $R^{2}$ (Table 3). It indicates an idea that others don't have to optimize these parameters for each machine, and the model is reasonably robust. The optimal hyperparameters are recommended to select in the stacked LSTM model when applied to another Linac. For a clinical routine, it is unnecessary to retrain the neural network each day with the daily acquired QC record.

If the stacked LSTM model works, it will be a great tool for medical physicists who are in charge of Linac's routine QC. It is possible that the model (LTSM) is overfitted resulting better performance compared to reference model (ARIMA). However, there exist some limitations in this study. Firstly, some hyperparameters correlate with each other and can result in different performances when optimized simultaneously rather than tuning.^28^ Secondly, due to prediction models being based on large learning‐phase data sets, the predictive models are not designed to detect large sudden one‐off jumps in data such as might be expected with a Linac component failure.

Predictive QC is more suited to detecting and predicting gradual drifts and failures that repeat at regular intervals. The present study results suggest that the approach of predictive QC based on MPC tests is feasible. Moreover, the stacked LSTM model is robust when applied to another radiotherapy machine with four QC items based on Quickcheck. In future work, the QC items of other types of radiotherapy machines will be applied in the stacked LSTM model, which should be fine‐tuned to obtain better predictive performance.

## 5 CONCLUSIONS

This study developed and evaluated a generalized stacked LSTM model for daily QC prediction. Moreover, the stacked LSTM model is robustness applied in another radiotherapy machine. This model has a better performance than the ARIMA model and can reduce the unscheduled Linac downtime and allows Linac performance parameters to be controlled within tolerances in the clinic.

## CONFLICT OF INTEREST

The authors have no conflict of interest to disclose.

## AUTHOR CONTRIBUTIONS

Study conception, design, data acquisition, and wrote paper: Min Ma. Drafted the manuscript: Jianrong Dai, Chenbin Liu, Ran Wei, and Bin Liang. Supervised the study: Jianrong Dai. Critical revision of the manuscript for important intellectual content: Min Ma, Chenbin Liu, Ran Wei, Bin Liang, and Jianrong Dai.

## Data Availability

The raw/processed data required to reproduce these findings cannot be shared at this time as the data also form part of an ongoing study.

## References

[1] Klein EE , Hanley J , Bayouth J , et al. Task Group 142 report: quality assurance of medical accelerators a. Med Phys. 2009;36:4197‐4212. 19810494 10.1118/1.3190392

[2] Li Q , Chan MF . Predictive time‐series modeling using artificial neural networks for Linac beam symmetry: an empirical study. Ann N Y Acad Sci. 2017;1387(1):84. 27627049 10.1111/nyas.13215 PMC5026311

[3] Siami‐Namini S , Tavakoli N , Siami Namin A . A comparison of ARIMA and LSTM in forecasting time series. Paper presented at: 2018 17th IEEE International Conference on Machine Learning and Applications (ICMLA); December 17–20, 2018; Orlando, FL.

[4] Tu Y‐H , Peng C‐C . An ARMA‐based digital twin for MEMS gyroscope drift dynamics modeling and real‐time compensation. IEEE Sens J. 2021;21(3):2712‐2724.

[5] Lee K , Lee C‐H , Kwak M‐S , et al. Analysis of multivariate longitudinal data using ARMA Cholesky and hypersphere decompositions. Comput Stat Data Anal. 2021;156:107144.

[6] Chakraborty D , Sanyal SK . Time‐series data optimized AR/ARMA model for frugal spectrum estimation in cognitive radio. Phys Commun. 2021;44:101252.

[7] Yu Y , Si X , Hu C , et al. A review of recurrent neural networks: LSTM cells and network architectures. Neural Comput. 2019;31(7):1235‐1270. 31113301 10.1162/neco_a_01199

[8] Hochreiter S , Schmidhuber J . Long short‐term memory. Neural Comput. 1997;9(8):1735‐1780. 9377276 10.1162/neco.1997.9.8.1735

[9] Puyati W , Khawne A , Barnes M , et al. Predictive quality assurance of a linear accelerator based on the machine performance check application using statistical process control and ARIMA forecast modeling. J Appl Clin Med Phys. 2020;21(8):73‐82. 10.1002/acm2.12917 PMC7484849 32543097

[10] Clivio A , Vanetti E , Rose S , et al. Evaluation of the machine performance check application for TrueBeam Linac. Radiat Oncol. 2015;10:97. 25896341 10.1186/s13014-015-0381-0 PMC4464869

[11] Barnes MP , Pomare D , Menk FW , et al. Evaluation of the truebeam machine performance check (MPC): OBI X‐ray tube alignment procedure. J Appl Clin Med Phys. 2018;19(6):68‐78. 10.1002/acm2.12445 PMC6236821 30178521

[12] Barnes MP , Greer PB . Evaluation of the TrueBeam machine performance check (MPC) beam constancy checks for flattened and flattening filter‐free (FFF) photon beams. J Appl Clin Med Phys. 2017;18(1):139‐150. 28291921 10.1002/acm2.12016 PMC5689878

[13] Barnes MP , Greer PB . Evaluation of the truebeam machine performance check (MPC): mechanical and collimation checks. J Appl Clin Med Phys. 2017;18(3):56‐66. 28419702 10.1002/acm2.12072 PMC5689839

[14] Barnes MP , Greer PB . Evaluation of the truebeam machine performance check (MPC) geometric checks for daily IGRT geometric accuracy quality assurance. J Appl Clin Med Phys. 2017;18(3):200‐206. 10.1002/acm2.12064 PMC5689847 28332342

[15] Gers FA , Schmidhuber J , Cummins F . Learning to forget: continual prediction with LSTM. Neural Comput. 2000;12(10):2451‐2471. 11032042 10.1162/089976600300015015

[16] Pascanu R , Mikolov T , Bengio Y . Understanding the exploding gradient problem. ArXiv. 2012. abs/1211.5063.

[17] Elman J . Finding structure in time. Cogn Sci. 1990;14:179‐211.

[18] Anastasi G , Bertholet J , Poulsen P , et al. Patterns of practice for adaptive and real‐time radiation therapy (POP‐ART RT) part I: intra‐fraction breathing motion management. Radiother Oncol. 2020;153:79‐87. 32585236 10.1016/j.radonc.2020.06.018 PMC7758783

[19] Li Y , Lin Z . Melody classifier with stacked‐LSTM. ArXiv. 2020. abs/2010.08123.

[20] Fern·ndez S , Graves A , Schmidhuber J . Sequence labelling in structured domains with hierarchical recurrent neural networks. Paper presented at: Proceedings of the 20th International Joint Conference on Artificial Intelligence; January 6‐12, 2007; Hyderabad, India.

[21] Zhang G . Time series forecasting using a hybrid ARIMA and neural network model. Neurocomputing. 2003;50:159‐175.

[22] Box G , Jenkins G . *Time Series Analysis, Forecasting and Control* . Wiley; 1970.

[23] Dhillon I , Ravikumar P , Tewari A . *Nearest Neighbor Based Greedy Coordinate Descent* . Curran Associates, Inc; 2011.

[24] Greff K , Srivastava R , KoutnÌk J , et al. LSTM: a search space odyssey. IEEE Trans Neural Networks Learn Syst. 2017;28:2222‐2232. 10.1109/TNNLS.2016.2582924 27411231

[25] Zaheer R , Shaziya H . A study of the optimization algorithms in deep learning. Paper presented at: 2019 Third International Conference on Inventive Systems and Control (ICISC); January 10‐11, 2019; Coimbatore, India.

[26] Wang S , Li C , Lim A . Why are the ARIMA and SARIMA not sufficient. ArXiv. 2019. abs/1904.07632.

[27] Chan MF , Li Q , Tang X , et al. Visual analysis of the daily QA results of photon and electron beams of a Trilogy Linac over a five‐year period. Int J Med Phys Clin Eng Radiat Oncol. 2015;4(4):290‐299. 27547595 10.4236/ijmpcero.2015.44035 PMC4989913

[28] Lin H , Shi C , Wang B , et al. Towards real‐time respiratory motion prediction based on long short‐term memory neural networks. Phys Med Biol. 2019;64(8):085010. 30917344 10.1088/1361-6560/ab13fa PMC6547821

[29] Varsamopoulos S , Bertels K , Almudever C . Designing neural network based decoders for surface codes 2018.

